# Management of malignant pleural mesothelioma—part 2: therapeutic approaches: Consensus of the Austrian Mesothelioma Interest Group (AMIG)

**DOI:** 10.1007/s00508-016-1036-3

**Published:** 2016-07-25

**Authors:** Mir Alireza Hoda, Thomas Klikovits, Madeleine Arns, Karin Dieckmann, Sabine Zöchbauer-Müller, Christian Geltner, Bernhard Baumgartner, Peter Errhalt, Barbara Machan, Wolfgang Pohl, Jörg Hutter, Josef Eckmayr, Michael Studnicka, Martin Flicker, Peter Cerkl, Walter Klepetko

**Affiliations:** 1 Division of Thoracic Surgery, Department of Surgery, Comprehensive Cancer Center, Medical University Vienna, Waehringer Guertel 18–20, 1090 Vienna, Austria; 2 Department of Pulmonology, Landesklinikum Hochegg, Hochegg, Austria; 3 Department of Radiation Oncology, Medical University Vienna, Vienna, Austria; 4 Department of Oncology, Medical University Vienna, Vienna, Austria; 5 Department of Pulmonology, Klinikum Klagenfurt, Klagenfurt, Austria; 6 Department of Pulmonology, Landeskrankenhaus Vöklabruck, Vöklabruck, Austria; 7 Department of Pulmonology, University Clinic Krems, Krems, Austria; 8 Rehabilitation Center Tobelbad, Allgemeine Unfallversicherungsanstalt, Tobelbad, Austria; 9 Department of Pulmonology, KH Hietzing, Karl Landsteiner Institute for Clinical and Experimental Pneumology, Vienna, Austria; 10 Department of Surgery, University Clinic Salzburg, Salzburg, Austria; 11 Department of Pulmonology, Landeskrankenhaus Wels, Wels, Austria; 12 Department of Pulmonology, University Clinic Salzburg, Salzburg, Austria; 13 Department of Pulmonology, Landeskrankenhaus Leoben, Leoben, Austria; 14 Department of Pulmonology, Landeskrankenhaus Hohenems, Hohenems, Austria

**Keywords:** Radiotherapy, Surgery, Multimodality treatment, Palliative treatment, Chemotherapy

## Abstract

Treatment of malignant pleural mesothelioma (MPM) depends on performance status of the patient, tumor stage, and histological differentiation. Chemotherapy (CHT) can be administered as first- and second-line treatment in unresectable MPM or as neoadjuvant or adjuvant treatment before or after surgery. A combination of an antifolate and platinum-based CHT is the only approved standard of care. Several targeted and immunotherapies are in evaluation and further studies are warranted to determine the therapeutic value of these new treatment options. Radiotherapy (RT) can be considered either as adjuvant treatment after surgery or for palliation of pain-related tumor growth. Recent data support the use of RT in a neoadjuvant setting. Macroscopic complete resection by pleurectomy/decortication (P/D) or extrapleural pneumonectomy (EPP) is indicated in selected patients with good performance status. Surgery should only be applied as part of a multimodality treatment (MMT) in combination with chemo- and/or radiotherapy. In a large number of cases, palliative attempts are needed to improve quality of life and to achieve symptom control.

## Introduction

Malignant pleural mesothelioma is a devastating disease and treatment is challenging. To date, there is no worldwide agreement on standardized treatment strategies. However, three treatment approaches—either combined or as single treatment—have evolved over the last decades: chemotherapy, radiotherapy, and surgery. Furthermore, intracavitary strategies such as intraoperative hyperthermic chemotherapy, photodynamic therapy and intrapleural gene and immunotherapy have been investigated as potential treatment options for local tumor control and prevention of recurrence of disease.

In the following we describe each approach and their combinations (multimodality protocols).

## Chemotherapy

### First-line treatment

The recommendations of a standard therapy with platinum-based CHT in combination with modern antifolates (pemetrexed or raltitrexed) are based on two randomized trials published in 2003 and 2005 [1, 2]. The median overall survival (OS) in the pemetrexed/cisplatin arm was 12.1 months versus 9.3 months in the control arm (*p* = 0.02). The median time to progression was significantly longer in the pemetrexed/cisplatin arm (5.7 months versus 3.9 months; *p* = 0.001). Although the cisplatin monotherapy was never compared to placebo in a randomized trial, these results enforced the recommendation for a combination chemotherapy. Carboplatin can be used as an acceptable alternative to cisplatin and may be better tolerated in elderly patients or patients with impaired functional status [3]. Other combination therapies as cisplatin + etoposide, methotrexate or interferon were tested but overall survival did not significantly improve compared to the combination of cisplatin with antifolates [4]. In principle, chemotherapy should be started as soon as possible after diagnosis. The duration of chemotherapy is 4–6 cycles of first-line therapy with cisplatin and pemetrexed. The experiences with this regimen derive from the treatment of NSCLC (Non-Small Cell Lung Cancer), where a therapy of four cycles is recommend usually. Six cycles are favored only in case of good overall response rates and lack of grade 3 and 4 toxicities.

### Second-line treatment

For second-line therapy, no general recommendations exist because little data is available in the literature. A phase III evaluation of pemetrexed monotherapy did not show any survival benefit compared to best supportive care in previously treated patients [5]. However, there is evidence of better survival with chemotherapy versus best supportive care [6]. Vinorelbine monotherapy has demonstrated a trend towards improved OS in a phase II trial, as it was shown in the first-line setting [7, 8]. Since there is currently no standard second-line therapy, it is recommended to enroll patients in clinical trials.

### Experimental approaches

Interleukins and interferons were tested in studies, as well as the application of targeted therapies with monoclonal antibodies. None of the following substances showed any survival benefit in several studies: thalidomide, gefitinib, erlotinib, or imatinib [9–14]. The first approach in combining bevacizumab with cisplatin/gemcitabine failed to show any survival benefit [10]. However, recently presented data of a randomized phase III trial combining cisplatin/pemetrexed with bevacizumab showed significantly improved progression-free and overall survival in previously untreated unresectable MPM patients [15]. The results of this study might lead to a new paradigm in standard treatment in these patients. Newer targeted therapies and immunotherapies as tremelimumab or ipilimumab are currently under investigation [16, 17]. Furthermore, the new PD-1 inhibiting agent pembrolizumab has shown promising results in antitumor activity and randomized trials are still ongoing [18].

## Radiotherapy

Currently, the most intensive treatment regime for pleural mesothelioma consists of a multimodality treatment combining chemotherapy, operation, and radiotherapy. However, there is still discussion whether patients actually benefit from the intensified treatment, as the long-term outcome is still poor independent of the combinations of treatment modalities. There are curative and palliative intentions to treat malignant pleura mesothelioma. In these settings radiotherapy can be performed as neoadjuvant, adjuvant, and palliative radiotherapy. The introduction of highly conformal radiotherapy technique (HCRT) improved dose delivery and target coverage in comparison to conventional 3‑dimensional conformal radiotherapy (3DCRT).

### Neoadjuvant radiotherapy

The concept of the preoperative neoadjuvant treatment of the hemithorax can be performed for patients with resectable pleural mesothelioma.

This concept (SMART) aims at inactivation of the tumor and improvement of its resectability [19, 20]. Patients are treated with a hypofractionated radiotherapy, which means the application of a higher dose of 5 Gy on 5 consecutive days with an integrated boost of 6 Gy to the macroscopic tumor. The target volume encompasses the whole lung including the visceral and parietal pleura. To reduce the high dose to the organs at risk as esophagus, liver, heart, or spinal cord, intensity-modulated radiotherapy technique (IMRT), VMAT (volumetric modulated arch therapy), or Tomotherapy is the treatment of choice. After radiotherapy an extrapleural pneumonectomy (EPP) has to be performed within 8–10 days after the end of radiotherapy.

### Adjuvant radiotherapy

**Fig. 1:**
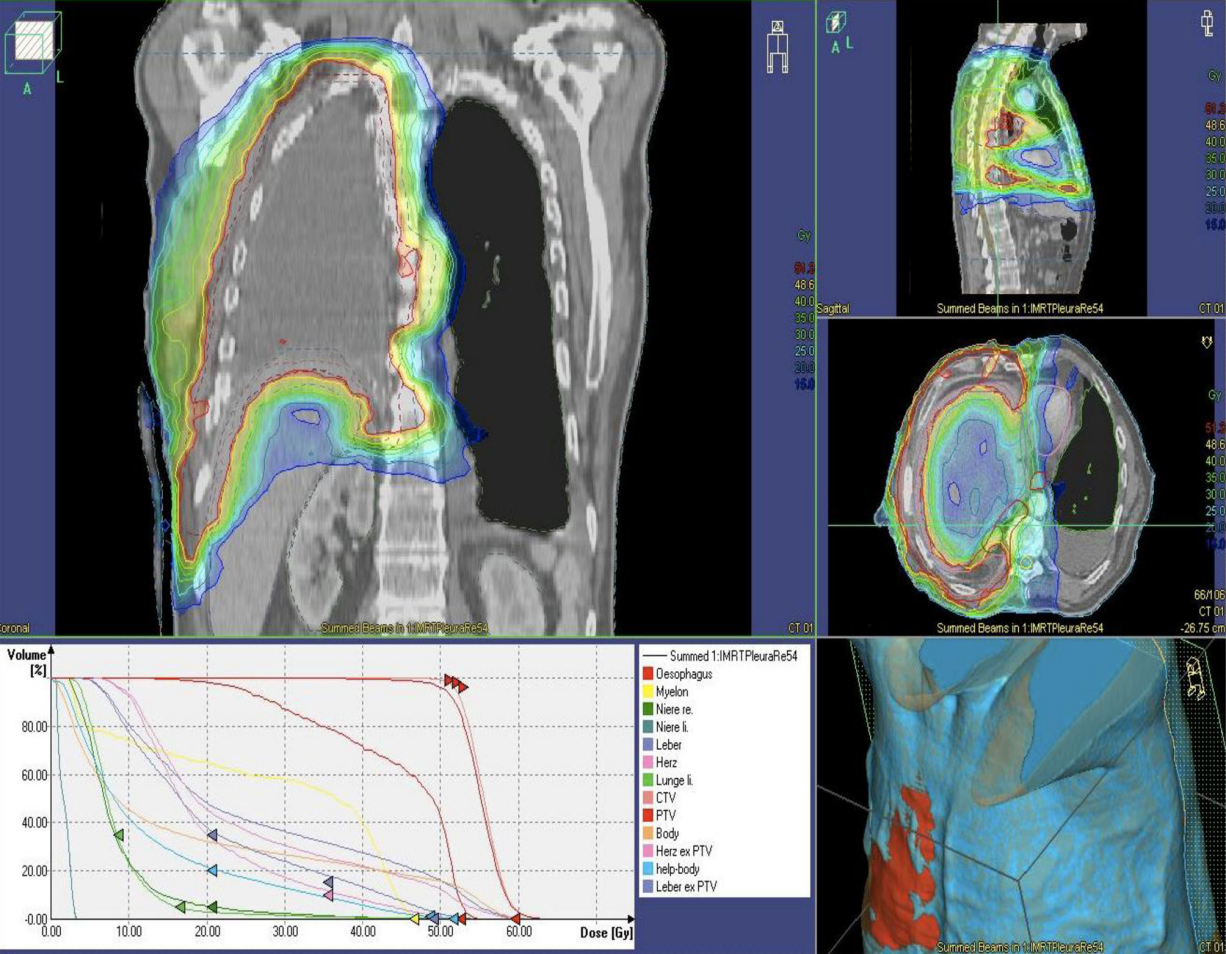
Postoperative situs after extrapleural pneumonectomy of the right lung and pleura. Intensity-modulated radiotherapy dose plan. Coronal, sagittal, and axial image of the isodose plan. Steep dose fall to the remaining left lung, liver, and kidney

Postoperative hemithoracic irradiation is regarded as part of the three modality treatment concept which combines chemotherapy, operation, and radiotherapy for resectable malignant pleural mesothelioma. The dose of 50–54 Gy should be applied with a once daily fraction of 1.8–2.0 Gy (Fig. 1).

Other fractionation schedules for 3D-conformal radiotherapy are recommended as 25 × 1.8 Gy (45 Gy) to the hemi thorax, followed by a boost onto the high-risk area with 7 × 1.8 Gy (12,6 Gy); or as another alternative 23 × 2 Gy (46 Gy) to the hemithorax, followed by 5 × 2 Gy boost which totals to 56–58 Gy for the high-risk area. In case of IMRT 26 × 1.75 Gy (45.5 Gy) with an integrated boost of 26 × 2.15 Gy (55.9 Gy) are possible [21].

Dose constrains to the organs at risk (OAR; as the non-involved lung, esophagus, spinal cord, liver or gastrointestinal organs, kidneys) have to be respected to avoid acute and long-term side effects as e. g. esophagitis and pneumonitis.

Radiotherapy should be initiated between 4–12 weeks after surgery depending on the wound healing process.

### Palliative radiotherapy

For inoperable pleural mesothelioma, a palliative radiotherapy treatment can be indicated with and without chemotherapy.

In case of inoperability, generally it is not possible to irradiate the whole hemithorax without damaging the non-affected lung tissue. Therefore, the tumor can be partially treated at regions where it causes pain or at regions of high risk infiltration of spinal cord to avoid side effects as paresis in the future. Treatment time should be short, a 3D conformal radiotherapy or IMRT can be applied with higher single dose (single dose of 3–4 Gy up to a total dose of 30–40 Gy), taking into account that parts of the tumor are not irradiated and included into the clinical target volume (CTV).

Symptom-based radiotherapy treatments of metastasis can be performed in all locations to control local pain or neurological problems. Start of treatment should be as soon as possible after presentation of the patient to avoid longer hospitalization and to improve quality of life. Doses of 3–4 Gy up to doses of 30 to 40 Gy or single doses of 8 Gy reduce the treatment time as much as possible.

Re-irradiation with a reduced dose can be a treatment option in case of local relapse taking into account the primarily applied radiation dose and the dose at the OAR.

## Surgery

Surgery for MPM can be applied in curative and palliative intention. Macroscopic complete resection (MCR) should be the goal of the respective surgical procedure. Recently, combined treatment modalities including radical surgery in terms of pleurectomy/decortication (P/D) or extrapleural pneumonectomy (EPP) are the most commonly used approaches for treating MPM in curative intention. Due to the difficulty of large randomized trials only institutional reports using different multimodality protocols and different surgical techniques are available to discuss whether P/D or EPP is the more appropriate procedure to obtain better long-term survival in relation to posttreatment quality of life.

After the IMIG 2012 meeting and much discussion on the role of surgery and the value of macroscopic complete resection the attendees agreed that (1) surgical macroscopic complete resection and control of micrometastatic disease play a vital role in the multimodality therapy of MPM, as it is the case for other solid malignancies. (2) Surgical cytoreduction is indicated when MCR is deemed achievable and (3) the type of surgery (EPP or P/D) depends on clinical factors and on individual surgical judgment and expertise [22].

Since the surgical technique of pleurectomy/decortication (P/D) in the treatment of MPM includes a variety of procedures with different clinical indications and therapeutic intents, the International Association for the Study of Lung Cancer (IASLC) and International Mesothelioma Interest Group (IMIG) have recently proposed a nomenclature for the different techniques [23]. Partial pleurectomy was defined as a cytoreductive procedure with partial removal of the visceral and/or parietal pleurae without the intention for macroscopic complete resection. P/D was defined as complete resection of the parietal and visceral pleurae and extended P/D as a technique with additional resection of the pericardium and diaphragm. A recent systematic review assessing the safety and efficacy of these techniques found that perioperative mortality and morbidity ranged from 0–11 % and 13–43 %, respectively. Median overall survival ranged from 7.1–31.7 months and disease-free survival ranged from 6–16 months [24]. A detailed analyses suggested similar perioperative mortality outcomes between different P/D techniques but a trend towards higher morbidity and length of hospitalization for patients who underwent extended P/D. However, overall and disease-free survival appeared to favor extended P/D compared to less aggressive techniques.

**Fig. 2:**
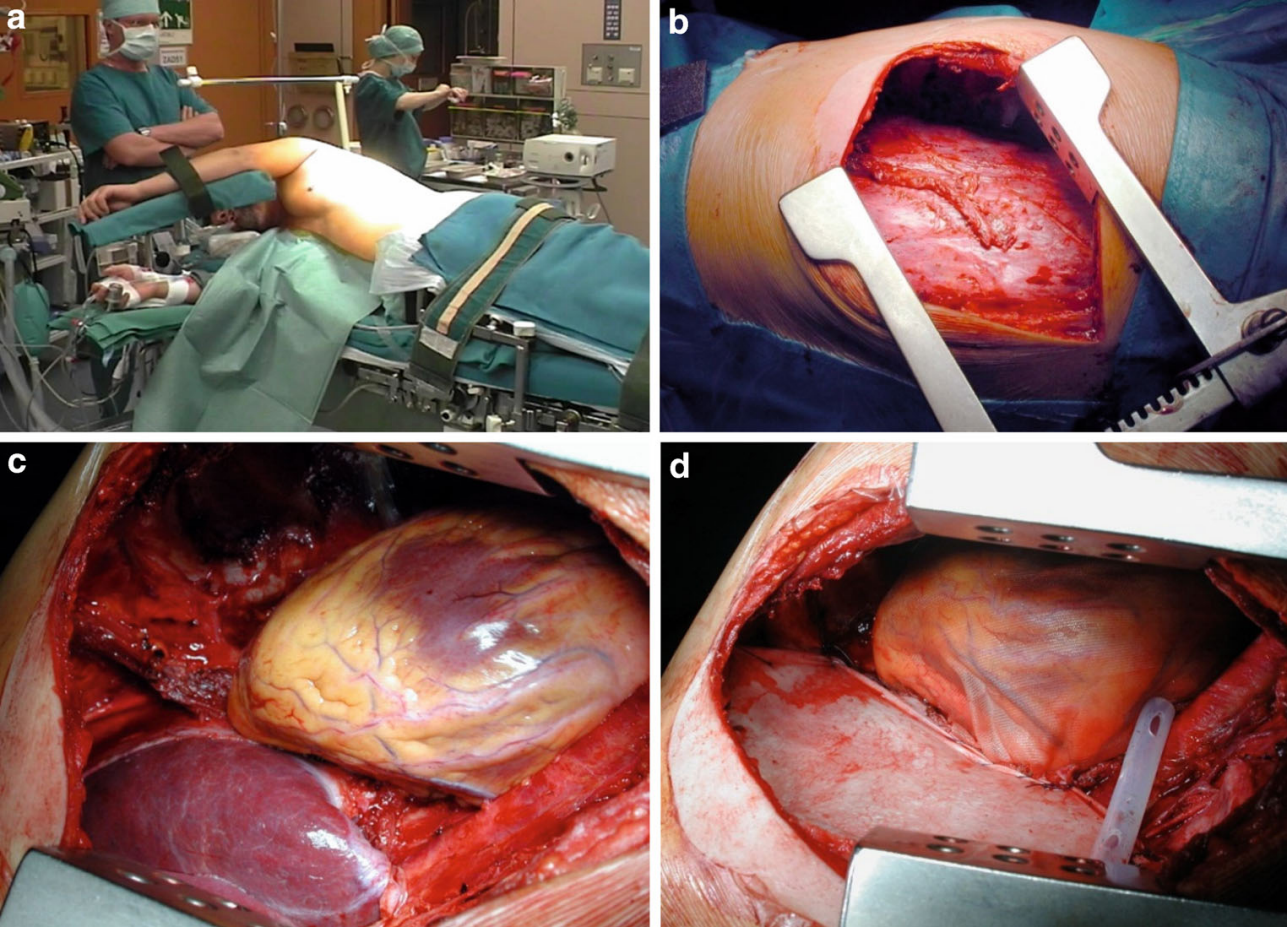
Surgical setting and intraoperative pictures of extrapleural pneumonectomy (EPP): **a** positioning of patient in OR; **b** situs after extrapleural mobilization of the lung; **c** lung, diaphragm, and pericardium have been removed; **d** situs after reconstruction of diaphragm and pericardium

Extrapleural pneumonectomy (EPP) is a widely standardized surgical procedure with en bloc resection of the parietal and visceral pleurae with the ipsilateral lung, pericardium and diaphragm (Fig. 2) [25]. The role of EPP within multimodality approaches was recently extensively discussed after the publication of the Mesothelioma and Radical Surgery (MARS I) trial which aimed to assess the clinical outcomes of patients who were randomly assigned to EPP or no EPP in the context of trimodal therapy [26]. The results of the study suggested that radical surgery in the form of EPP within trimodality therapy offers no benefit and possibly harmed patients. However, the MARS I trial was designed to assess the feasibility of such a study and not to evaluate the risks and benefits of EPP. In order to sufficiently answer this question an actual number of 670 patients to identify a significant survival benefit would have been needed. A recent systematic review on the safety and efficacy of EPP for patients with MPM including 58 studies reported that median overall survival varied from 9.4–27.5 months, and 1‑, 2‑, and 5‑year survival rates ranged from 36–83 %, 5–59 %, and 0–24 %, respectively. Overall perioperative mortality rates were reported to range from 0–11.8 %, and the perioperative morbidity rates from 22–82 % [27]. Another recent retrospective evaluation of 3 high volume institutions including 251 patients completing EPP after platin-based induction treatment reported a 30-day mortality of 5 % and perioperative complication rate of 30 % [28]. However, according to the latest ERS/ESTS guidelines, EPP in a curative intent should only be performed at high volume centers in terms of a clinical study and within a multimodal treatment algorithm [29].

## Multimodality treatment

Since several studies have shown that surgical treatment alone offers dismal prognosis only, multimodality approaches including chemo- and radiotherapy have been suggested in order to improve survival. Regarding the exact sequence of treatment modalities there are no randomized trials available to answer the question if chemo-, radio-, or combined chemo/radiotherapy should be used as induction treatment before surgery. With regard to EPP trimodality approaches, many high volume centers include chemotherapy as induction treatment followed by surgery and then by hemithoracic radiotherapy [30, 31]. The particular advantage of chemotherapy before surgery is a possible reduction of tumor load and downstaging, which may lead to a better understanding of the individual tumor biology and a more accurate patient selection. The possible effects of induction chemotherapy have recently been studied in a prospective multicenter trial including 61 patients being considered surgical candidates at diagnosis. Forty-five patients (74 %) underwent EPP and in 37 patients (61 %) the resection was complete. Postoperative radiotherapy was initiated in 36 patients. The median survival of all patients was 19.8 months. For the 45 patients undergoing EPP, the median survival was 23 months [32]. Another multimodal approach was recently introduced by the Toronto group which is currently under investigation in the Surgery for Mesothelioma After Radiation Therapy (SMART) trial. A short accelerated course of high-dose hemithoracic intensity-modulated radiation therapy (IMRT) is administered followed by EPP [19]. In all, 25 patients with resectable clinical stage T1-3N0M0 histologically proven, previously untreated MPM were included. Initial results revealed a cumulative 3‑year survival of 84 % in epithelial subtypes compared with 13 % in biphasic subtypes. Furthermore, the results suggested that this protocol is feasible without elevated perioperative morbidity and mortality. However, further prospective trials are needed in order to investigate the value of different multimodality strategies in the treatment of MPM.

### Intracavitary treatment

Local recurrence is a frequent problem even after macroscopic complete resection and occurs in up to 60 % of the cases [31]. This may be caused by the infiltrative growing pattern of MPM to the surrounding tissue and the inability to achieve 100 % tumor-free margins (R0 resection) after resection. Recently, additional treatment approaches such as intracavitary chemo-, immuno-, or photodynamic therapy have been suggested to postoperatively secure resection margins and to lower the risk of local recurrence.

Intracavitary photodynamic therapy (PDT) combines a nontoxic photosensitizing compound with visible light and can be delivered intraoperatively after P/D or EPP [33]. The concept of PDT combined with radical pleurectomy was recently studied in 38 patients in predominantly stage III/IV disease with a median survival of 31.7 months for all 38 patients and 41.2 months for the 31/38 (82 %) patients with epithelial subtypes despite a median progression-free survival (PFS) of 9.6 and 15.1 only [34].

It has been proven that hyperthermic intraoperative chemotherapy (HIOC) is able to deliver a higher local dose chemotherapy to the resected surface with decreased toxicity compared to systemic therapy [35, 36]. The feasibility and effects of HIOC have recently been investigated in several phase II trials and extended interval to recurrence and overall survival have been reported in a group of patients with epithelioid MPM and low risk factors [37–39]. Other intracavitary concepts of binding cytotoxic (or other agents) to a fibrin carrier are currently evaluated in phase I and II trials. The concept of localized intracavitary cisplatin–fibrin chemotherapy after MCR is evaluated in a phase IIa trial to assess safety and toxicity of the treatment (NCT01644994 Influence Meso) [40].

### Patient selection and prognostic factors

Taken all this information together, patients undergoing MCR for MPM must be meticulously selected in order to outweigh the benefits compared to the potential risks. Several studies have been published different prognostic factors and biomarkers for stratifying patients into different prognostic groups. A recent systematic review of prognostic factors and patient selection reported that those with non-epithelial MPM and nodal involvement have consistently demonstrated to have a worse prognosis after EPP [41]. Furthermore, several blood biomarkers have recently been investigated to identify patients who will benefit from surgical treatment. Among these, preinterventional C‑reactive protein (CRP) was found to predict benefit from multimodality treatment including radical surgery [42]. Patients with elevated CRP levels had a significantly shorter overall survival compared with those with normal CRP, which was confirmed in multivariate analyses. In other recent studies biomarkers such as fibrinogen, albumin, or MPM specific prognostic scores (currently under investigation, unpublished data) were reported to have prognostic impact and could be useful in clinical decision making [43, 44]. Moreover, a recent phase II study reported that progression-free survival could be useful as a prognostic marker for overall survival rather than response rates to chemotherapy. Patient data from 10 European Organisation for Research and Treatment of Cancer (EORTC) studies of first-line chemotherapy in MPM were pooled and progression-free survival at 18 weeks after randomization was strongly correlated and discriminated patients with better OS from the poorer prognosis patients [45].

Finally, there is not much data available on the quality of life (QoL) during and after treatment. A recent study has included QoL data in their institutional report and found a superiority of P/D over EPP in QoL after 6 and 12 months [46].Thus, the MARS trial reported that median quality of life scores were lower in the EPP group than the no EPP group; however, no significant differences between groups were reported in the quality of life analyses [26].

However, since MPM is a very heterogeneous disease with variability of clinical symptoms, stage, histology, tumor burden, and biological behavior, a multidisciplinary discussion of every patient considering age, performance status, and individual prognosis should be mandatory. All currently available therapeutic modalities in MPM are summerized in Table 1.

## Palliative surgical procedures and symptom control

Usually, MPM is diagnosed at an advanced stage and symptom control is an important part of every curative and palliative therapy attempt [47]. Common symptoms are dyspnea due to pleural effusion (in early stages) or lung encasement by pleural thickening (in later stages), weight loss, cough, and chest pain caused by invasion of the thoracic wall. When dyspnea is caused by pleural effusion early drainage for symptom control is recommended followed by pleurodesis at first relapse [29]. Sterile talc powder is the preferred sclerosing agent for pleurodesis and can be installed through a chest drainage or during diagnostic VATS biopsy if the lung completely comes to expansion [29, 47]. Recurrent pleural effusions, especially in case of an entrapped lung can become more difficult to manage and indwelling pleural catheters may be the most practical way to manage recurrent pleural effusions [48, 49]. Furthermore, VATS partial pleurectomy (VATS-PP) has been suggested to improve symptom control and survival. Recently, the MesoVATS trial has randomized MPM patients to undergo VATS pleurectomy vs. talc pleurodesis via an indwelling intercostal chest drain or via thoracoscopy [50]. VATS-PP did not significantly improve survival and talc pleurodesis was considered to be preferable due to fewer complications and shorter hospital stay. However, VATS-PP significantly improved control of recurrent pleural effusions in the first 6 months after the procedure and improved QoL for 12 months.

## Summary

**Fig. 3:**
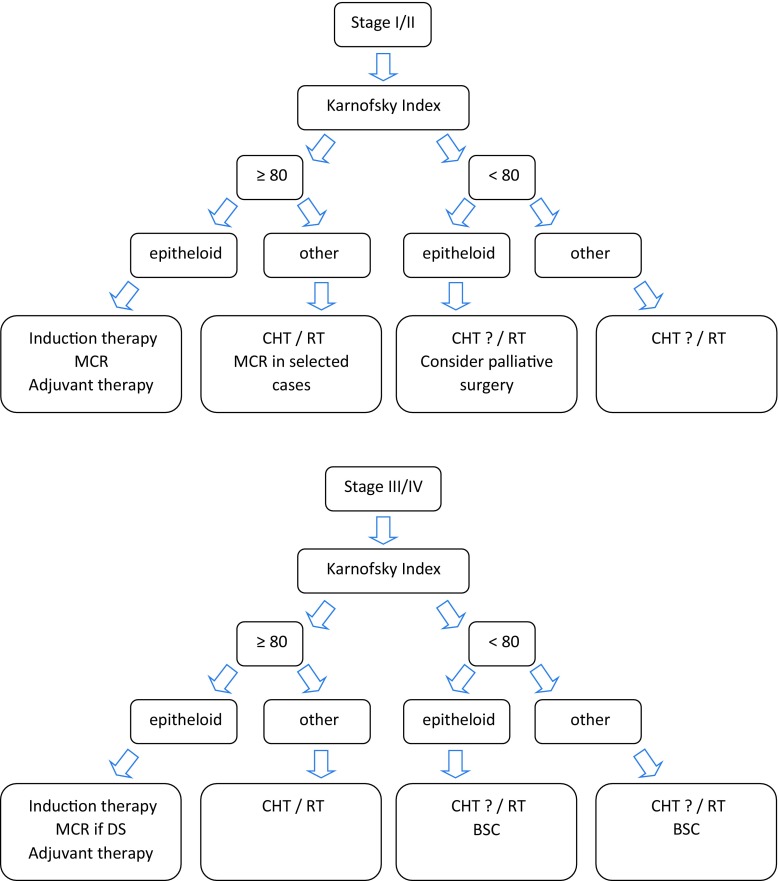
Proposed therapeutic algorithm in malignant pleural mesothelioma. *MCR* macroscopic complete resection, *CHT* chemotherapy, *RT* radiotherapy, *DS* downstaging, *BSC* best supportive care

At the time present, no worldwide accepted surgical therapy exists and the choice of procedure depends on tumor stage, clinical presentation, and especially on institutional expertise and preferences. Pooling of MPM patients in specialized centers is mandatory to improve results of surgical treatment within multimodality protocols. A proposed, stage-dependent treatment algorithm is depicted in Fig. 3.

**Table 1:** Main therapeutic approaches for the treatment of MPM

*Chemotherapy*	Neoadjuvant/adjuvant/palliative *Intraoperative:* HITOC/photodynamic therapy
New approaches	Targeted therapy Immunotherapy
*Radiotherapy*	Adjuvant/neoadjuvant/palliative Conventional RT/IMRT
*Surgery*	*Macroscopic complete resection:* Extrapleural pneumonectomy Extended pleurectomy/decortication (P/D) *Palliative approaches:* Partial pleurectomy/decortication (P/D) Talc pleurodesis

*HITOC* hyperthermic intraoperative chemotherapy, *RT* radiotherapy, *IMRT* intensity modullated radiotherapy

## References

[1] Vogelzang NJ, Rusthoven JJ, Symanowski J, Denham C, Kaukel E, Ruffie P (2003). Phase III study of pemetrexed in combination with cisplatin versus cisplatin alone in patients with malignant pleural mesothelioma. J Clin Oncol.

[2] van Meerbeeck JP, Gaafar R, Manegold C, van Klaveren RJ, van Marck EA, Vincent M (2005). Randomized phase III study of cisplatin with or without raltitrexed in patients with malignant pleural mesothelioma: an intergroup study of the European Organisation for Research and Treatment of Cancer Lung Cancer Group and the National Cancer Institute of Canada. J Clin Oncol.

[3] Santoro A, O’Brien ME, Stahel RA, Nackaerts K, Baas P, Karthaus M (2008). Pemetrexed plus cisplatin or pemetrexed plus carboplatin for chemonaïve patients with malignant pleural mesothelioma: results of the International Expanded Access Program. J Thorac Oncol.

[4] Berghmans T, Paesmans M, Lalami Y, Louviaux I, Luce S, Mascaux C (2002). Activity of chemotherapy and immunotherapy on malignant mesothelioma: a systematic review of the literature with meta-analysis. Lung Cancer.

[5] Baas P, Fennell D, Kerr KM, Van Schil PE, Haas RL, Peters S (2015). Malignant pleural mesothelioma: ESMO Clinical Practice Guidelines for diagnosis, treatment and follow-up. Ann Oncol.

[6] Manegold C, Symanowski J, Gatzemeier U, Reck M, von Pawel J, Kortsik C (2005). Second-line (post-study) chemotherapy received by patients treated in the phase III trial of pemetrexed plus cisplatin versus cisplatin alone in malignant pleural mesothelioma. Ann Oncol.

[7] Stebbing J, Powles T, McPherson K, Shamash J, Wells P, Sheaff MT (2009). The efficacy and safety of weekly vinorelbine in relapsed malignant pleural mesothelioma. Lung Cancer.

[8] Zauderer MG, Kass SL, Woo K, Sima CS, Ginsberg MS, Krug LM (2014). Vinorelbine and gemcitabine as second- or third-line therapy for malignant pleural mesothelioma. Lung Cancer.

[9] Buikhuisen WA, Burgers JA, Vincent AD, Korse CM, van Klaveren RJ, Schramel FM (2013). Thalidomide versus active supportive care for maintenance in patients with malignant mesothelioma after first-line chemotherapy (NVALT 5): an open-label, multicentre, randomised phase 3 study. Lancet Oncol.

[10] Kindler HL, Karrison TG, Gandara DR, Lu C, Krug LM, Stevenson JP (2012). Multicenter, double-blind, placebo-controlled, randomized phase II trial of gemcitabine/cisplatin plus bevacizumab or placebo in patients with malignant mesothelioma. J Clin Oncol.

[11] Baas P, Boogerd W, Dalesio O, Haringhuizen A, Custers F, van Zandwijk N (2005). Thalidomide in patients with malignant pleural mesothelioma. Lung Cancer.

[12] Govindan R, Kratzke RA, Herndon JE, Niehans GA, Vollmer R, Watson D (2005). Gefitinib in patients with malignant mesothelioma: a phase II study by the Cancer and Leukemia Group B. Clin Cancer Res.

[13] Garland LL, Rankin C, Gandara DR, Rivkin SE, Scott KM, Nagle RB (2007). Phase II study of erlotinib in patients with malignant pleural mesothelioma: a Southwest Oncology Group Study. J Clin Oncol.

[14] Mathy A, Baas P, Dalesio O, van Zandwijk N (2005). Limited efficacy of imatinib mesylate in malignant mesothelioma: a phase II trial. Lung Cancer.

[15] Zalcman G, Mazieres J, Margery J, Greillier L, Audigier-Valette C, Moro-Sibilot D (2015). Bevacizumab for newly diagnosed pleural mesothelioma in the Mesothelioma Avastin Cisplatin Pemetrexed Study (MAPS): a randomised, controlled, open-label, phase 3 trial. Lancet.

[16] Remon J, Reguart N, Corral J, Lianes P (2015). Malignant pleural mesothelioma: new hope in the horizon with novel therapeutic strategies. Cancer Treat Rev.

[17] Calabrò L, Morra A, Fonsatti E, Cutaia O, Fazio C, Annesi D (2015). Efficacy and safety of an intensified schedule of tremelimumab for chemotherapy-resistant malignant mesothelioma: an open-label, single-arm, phase 2 study. Lancet Respir Med.

[18] Marcq E, Pauwels P, van Meerbeeck JP, Smits EL (2015). Targeting immune checkpoints: New opportunity for mesothelioma treatment?. Cancer Treat Rev.

[19] Cho BC, Feld R, Leighl N, Opitz I, Anraku M, Tsao MS (2014). A feasibility study evaluating Surgery for Mesothelioma After Radiation Therapy: the “SMART” approach for resectable malignant pleural mesothelioma. J Thorac Oncol.

[20] Clive AO, Wilson P, Taylor H, Morley AJ, de Winton E, Panakis N (2015). Protocol for the surgical and large bore procedures in malignant pleural mesothelioma and radiotherapy trial (SMART Trial): an RCT evaluating whether prophylactic radiotherapy reduces the incidence of procedure tract metastases. BMJ Open.

[21] Cao C, Tian D, Manganas C, Matthews P, Yan TD (2012). Systematic review of trimodality therapy for patients with malignant pleural mesothelioma. Ann Cardiothorac Surg.

[22] Rusch V, Baldini EH, Bueno R, De Perrot M, Flores R, Hasegawa S (2012). The role of surgical cytoreduction in the treatment of malignant pleural mesothelioma: meeting summary of the International Mesothelioma Interest Group Congress, September 11–14. Mass J Thorac Cardiovasc Surg.

[23] Rice D, Rusch V, Pass H, Asamura H, Nakano T, Edwards J (2011). Recommendations for uniform definitions of surgical techniques for malignant pleural mesothelioma: a consensus report of the international association for the study of lung cancer international staging committee and the international mesothelioma interest group. J Thorac Oncol.

[24] Cao C, Tian DH, Pataky KA, Yan TD (2013). Systematic review of pleurectomy in the treatment of malignant pleural mesothelioma. Lung Cancer.

[25] Sugarbaker DJ, Jaklitsch MT, Bueno R, Richards W, Lukanich J, Mentzer SJ (2004). Prevention, early detection, and management of complications after 328 consecutive extrapleural pneumonectomies. J Thorac Cardiovasc Surg.

[26] Treasure T, Lang-Lazdunski L, Waller D, Bliss JM, Tan C, Entwisle J (2011). Extra-pleural pneumonectomy versus no extra-pleural pneumonectomy for patients with malignant pleural mesothelioma: clinical outcomes of the Mesothelioma and Radical Surgery (MARS) randomised feasibility study. Lancet Oncol.

[27] Cao CQ, Yan TD, Bannon PG, McCaughan BC (2010). A systematic review of extrapleural pneumonectomy for malignant pleural mesothelioma. J Thorac Oncol.

[28] Lauk O, Hoda MA, de Perrot M, Friess M, Klikovits T, Klepetko W (2014). Extrapleural Pneumonectomy After Induction Chemotherapy: Perioperative Outcome in 251 Mesothelioma Patients From Three High-Volume Institutions. Ann Thorac Surg.

[29] Scherpereel A, Astoul P, Baas P, Berghmans T, Clayson H, de Vuyst P (2010). Guidelines of the European Respiratory Society and the European Society of Thoracic Surgeons for the management of malignant pleural mesothelioma. Eur Respir J.

[30] Sugarbaker DJ, Garcia JP (1997). Multimodality therapy for malignant pleural mesothelioma. Chest.

[31] Flores RM, Pass HI, Seshan VE, Dycoco J, Zakowski M, Carbone M (2008). Extrapleural pneumonectomy versus pleurectomy/decortication in the surgical management of malignant pleural mesothelioma: results in 663 patients. J Thorac Cardiovasc Surg.

[32] Weder W, Stahel RA, Bernhard J, Bodis S, Vogt P, Ballabeni P (2007). Multicenter trial of neo-adjuvant chemotherapy followed by extrapleural pneumonectomy in malignant pleural mesothelioma. Ann Oncol.

[33] Friedberg JS (2009). Photodynamic therapy as an innovative treatment for malignant pleural mesothelioma. Semin Thorac Cardiovasc Surg.

[34] Friedberg JS, Culligan MJ, Mick R, Stevenson J, Hahn SM, Sterman D (2012). Radical pleurectomy and intraoperative photodynamic therapy for malignant pleural mesothelioma. Ann Thorac Surg.

[35] Ried M, Potzger T, Braune N, Diez C, Neu R, Sziklavari Z (2013). Local and systemic exposure of cisplatin during hyperthermic intrathoracic chemotherapy perfusion after pleurectomy and decortication for treatment of pleural malignancies. J Surg Oncol.

[36] Sugarbaker PH, Stuart OA, Eger C (2012). Pharmacokinetics of Hyperthermic Intrathoracic Chemotherapy following Pleurectomy and Decortication. Gastroenterol Res Pract.

[37] Tilleman TR, Richards WG, Zellos L, Johnson BE, Jaklitsch MT, Mueller J (2009). Extrapleural pneumonectomy followed by intracavitary intraoperative hyperthermic cisplatin with pharmacologic cytoprotection for treatment of malignant pleural mesothelioma: a phase II prospective study. J Thorac Cardiovasc Surg.

[38] Richards WG, Zellos L, Bueno R, Jaklitsch MT, Jänne PA, Chirieac LR (2006). Phase I to II study of pleurectomy/decortication and intraoperative intracavitary hyperthermic cisplatin lavage for mesothelioma. J Clin Oncol.

[39] Sugarbaker DJ, Gill RR, Yeap BY, Wolf AS, DaSilva MC, Baldini EH (2013). Hyperthermic intraoperative pleural cisplatin chemotherapy extends interval to recurrence and survival among low-risk patients with malignant pleural mesothelioma undergoing surgical macroscopic complete resection. J Thorac Cardiovasc Surg.

[40] Opitz I (2014). Management of malignant pleural mesothelioma-The European experience. J Thorac Dis.

[41] Cao C, Yan TD, Bannon PG, McCaughan BC (2011). Summary of prognostic factors and patient selection for extrapleural pneumonectomy in the treatment of malignant pleural mesothelioma. Ann Surg Oncol.

[42] Ghanim B, Hoda MA, Winter MP, Klikovits T, Alimohammadi A, Hegedus B (2012). Pretreatment serum C‑reactive protein levels predict benefit from multimodality treatment including radical surgery in malignant pleural mesothelioma: a retrospective multicenter analysis. Ann Surg.

[43] Ghanim B, Hoda MA, Klikovits T, Winter MP, Alimohammadi A, Grusch M (2014). Circulating fibrinogen is a prognostic and predictive biomarker in malignant pleural mesothelioma. Br J Cancer.

[44] Yao ZH, Tian GY, Yang SX, Wan YY, Kang YM, Liu QH (2014). Serum albumin as a significant prognostic factor in patients with malignant pleural mesothelioma. Tumour Biol.

[45] Hasan B, Greillier L, Pallis A, Menis J, Gaafar R, Sylvester R (2014). Progression free survival rate at 9 and 18 weeks predict overall survival in patients with malignant pleural mesothelioma: An individual patient pooled analysis of 10 European Organisation for Research and Treatment of Cancer Lung Cancer Group studies and an independent study validation. Eur J Cancer.

[46] Rena O, Casadio C (2012). Extrapleural pneumonectomy for early stage malignant pleural mesothelioma: a harmful procedure. Lung Cancer.

[47] BTSSoC C (2007). BTS statement on malignant mesothelioma in the UK, 2007. Thorax.

[48] Lee YC, Fysh ET (2011). Indwelling pleural catheter: changing the paradigm of malignant effusion management. J Thorac Oncol.

[49] Suzuki K, Servais EL, Rizk NP, Solomon SB, Sima CS, Park BJ (2011). Palliation and pleurodesis in malignant pleural effusion: the role for tunneled pleural catheters. J Thorac Oncol.

[50] Rintoul RC, Ritchie AJ, Edwards JG, Waller DA, Coonar AS, Bennett M (2014). Efficacy and cost of video-assisted thoracoscopic partial pleurectomy versus talc pleurodesis in patients with malignant pleural mesothelioma (MesoVATS): an open-label, randomised, controlled trial. Lancet.

